# Achieving Cardiovascular Risk Management Goals and Patient Quality of Life

**DOI:** 10.3390/jcdd11020045

**Published:** 2024-01-31

**Authors:** Agata Kosobucka-Ozdoba, Łukasz Pietrzykowski, Piotr Michalski, Jakub Ratajczak, Klaudyna Grzelakowska, Michał Kasprzak, Jacek Kubica, Aldona Kubica

**Affiliations:** 1 Department of Cardiac Rehabilitation and Health Promotion, Collegium Medicum in Bydgoszcz, Nicolaus Copernicus University in Toruń, 85-094 Bydgoszcz, Poland; a.kosobucka@cm.umk.pl (A.K.-O.); piotr.michalski@cm.umk.pl (P.M.); jakub.ratajczak@cm.umk.pl (J.R.); akubica@cm.umk.pl (A.K.); 2 Department of Cardiology and Internal Medicine, Collegium Medicum in Bydgoszcz, Nicolaus Copernicus University in Toruń, 85-094 Bydgoszcz, Poland; klaudyna.grzelakowska@gmail.com (K.G.); michal.kasprzak@cm.umk.pl (M.K.); jkubica@cm.umk.pl (J.K.)

**Keywords:** quality of life, risk factors, ischemic heart disease

## Abstract

(1) Background: Eliminating or reducing the severity of modifiable risk factors of cardiovascular disease (CVD) and undertaking health-promoting behaviors is the basis for prevention. (2) Methods: This study included 200 subjects without a history of CVD, aged 18 to 80 years, who had been diagnosed with hypertension, hypercholesterolemia, or diabetes 6 to 24 months before study enrolment. (3) Results: The median 10-year CV risk assessed by the SCORE2 and SCORE2-OP algorithms was 3.0 (IQR 1.5–7.0). An increase in mean cardiovascular risk in the range from low and moderate to very high was associated with a decrease in quality of life both in individual subscales and the overall score. The median number of controlled risk factors was 4.0 (IQR 3.0–5.0). As the mean number of controlled risk factors increased, the quality of life improved in both of HeartQoL questionnaire subscales (emotional *p* = 0.0018; physical *p* = 0.0004) and the overall score (global *p* = 0.0001). The median number of reported health-promoting behaviors undertaken within 3 years before study enrolment was 3.0 (IQR 2.0–4.0). The highest quality of life in each of the studied dimensions was found in people who reported undertaking three health-promoting behaviors. (4) Conclusions: Controlling CVD risk factors and undertaking health-promoting behaviors has a positive impact on the quality of life of patients without a history of atherosclerotic CVD.

## 1. Introduction

The achievement of treatment goals for the control of risk factors is the basis of cardiovascular disease (CVD) treatment [1,2] and prevention [3]. Assessing the quality of life of chronically ill patients is considered a necessary element in a comprehensive health assessment. It can and should serve as an additional source of information on the physical, mental, and social well-being of the patient [3,4,5,6]. Higher quality of life is associated with more effective control of risk factors, better functioning, and prognosis [4,5,6,7,8]. Little is known about the relationship between the efficacy of cardiovascular risk factors’ management and patients’ quality of life, particularly those without clinically evident atherosclerotic cardiovascular disease. Therefore, we undertook a study of the relationship between the achievement of therapeutic targets for cardiovascular risk factors and both the health-promoting behaviors undertaken and the quality of life in people without diagnosed CVD.

## 2. Materials and Methods

This study included 200 subjects without a history of CVD, aged 18 to 80 years, who had been diagnosed with hypertension, hypercholesterolemia, or diabetes 6 to 24 months before study enrolment. The characteristics of the study group are presented in Table 1 and Table 2.

This study was designed as a multi-center prospective registry, approved by the Ethics Committee of Nicolaus Copernicus University in Toruń, Collegium Medicum in Bydgoszcz (study approval reference number KB 586/2017). Individual patients were invited to this study after prior identification of inclusion criteria fulfillment based on medical records in primary care facilities. In compliance with the principles of good clinical practice and the Declaration of Helsinki, each patient gave written informed consent to participate in this study. During the only visit scheduled for this study, venous blood samples were collected and anthropometric measurements (height, body weight, waist circumference) were taken. Measures of cardiovascular (CV) risk factors were assessed: arterial blood pressure, body mass index (BMI), waist circumference, physical activity, smoking status, serum concentrations of total cholesterol (TC), high-density lipoprotein cholesterol (HDL-C), non-high-density lipoprotein cholesterol (non-HDL-C), low-density lipoprotein cholesterol (LDL-C), triglycerides (TG), and fasting plasma glucose. A history of health-promoting behaviors during the 3 years before inclusion in this study was collected: dietary changes, weight reduction, increased physical activity, smoking cessation, and decreased alcohol consumption. Furthermore, cardiovascular risk was assessed using the Systematic Coronary Risk Evaluation 2 (SCORE2) and Systematic Coronary Risk Evaluation 2—Older Persons (SCORE2-OP) algorithms. In addition, quality of life was assessed using the HeartQoL questionnaire.

Arterial blood pressure was measured twice in a sitting position using semi-automatic sphygmomanometers. Diagnosis and classification of arterial hypertension were performed based on the current criteria of the European Society of Cardiology [9].

A current smoking status was verified by assessing the concentration of carbon monoxide in exhaled air (Bedfont Scientific Micro+ Smokerlyzer monitor). A result of >10 ppm was considered indicative of active smoking.

Measurement of serum TC, HDL-C, non-HDL-C, LDL-C, TG, and fasting blood glucose in venous blood samples was performed using the Alinity ci-series analyzer (Abbott, Wiesbaden, Germany).

Physical activity was assessed based on patients’ answers to the following question: ‘Which of the following terms best describes your non-professional activity?’ with 4 possible answers: (1) ‘I do not engage in any physical activity other than professional work’; (2) ‘Most of the time only light physical activity’; (3) ‘Intense physical activity at least 20 min 1–2 times a week’; (4) ‘20 min of intense physical activity more often than twice a week’. Responses 3 and 4 were considered to be the appropriate level of physical activity.

The risk factors within which the therapeutic target was achieved were defined as a controlled risk factors using the following criteria:

1. Ideal blood pressure: systolic blood pressure <140 mmHg and diastolic blood pressure <90 mmHg;
2. Ideal BMI: BMI 20.0–24.9 kg/m^2^; however, patients with BMI < 20.0 kg/m^2^ were not included in the analysis of uncontrolled risk factors with regards to BMI (11 patients);
3. Ideal waist circumference: <80 cm for women and <94 cm for men;
4. Regular physical activity: intense physical activity for 20 min or more at least 1–2 times a week;
5. Non-smoker: the declared status of a non-smoker was objectively confirmed by a concentration of carbon monoxide in exhaled air ≤10 ppm;
6. Normal LDL-C: <2.6 mmol/L (<100 mg/dL);
7. Normal TG: <1.7 mmol/L (<150 mg/dL);
8. Normal fasting glucose: <100 mg/dL (<5.6 mmol/L).

The health-promoting behaviors that the subjects undertook over a period of 3 years were assessed based on subjective declarations. Respondents answered the question ‘Have you used any of these methods in recent years to reduce your risk of developing ischemic heart disease?’:

1. Dietary changes (decrease in salt intake, decrease in fat intake, change in the type of fat consumed, decrease in meals’ caloric content, increase in fruit and vegetable intake, increase in fish intake, increase in fatty fish intake, decrease in sugar intake);
2. Weight reduction (with diet, by participating in regular physical exercise, using weight loss medications);
3. Increased physical activity (as part of a professionally established exercise program, by participating in fitness club activities, increasing physical activity on most days of the week, jogging or hiking);
4. Smoking cessation (abstinence, reduction in the number of cigarettes smoked, advice in a smoking cessation clinic, use of nicotine replacement therapy);
5. Decreased alcohol consumption.

For each of the methods, the respondent could answer: (1) ‘No’, (2) ‘Yes’, or (3) ‘I don’t know’. Only the answer ‘Yes’ was considered to indicate a health-promoting behavior in individual categories.

CV risk was assessed using the SCORE2 and SCORE2-OP algorithms according to the guidelines of the European Society of Cardiology with adjusted norms for Poland as a high-risk country based on SCORE2 and SCORE2-OP risk regions [10,11]. Using the SCORE2 and SCORE2-OP algorithms, the 10-year risk of CV death in apparently healthy subjects was calculated taking into account gender, age, non-HDL cholesterol concentration, systolic blood pressure, and smoking status. Cardiovascular risk was expressed as a percentage and then defined as low or moderate, high, or very high based on ESC guidelines [10,11]. Patients with diagnosed diabetes (n = 38) were excluded from CV risk assessment due to the lack of complete data required for assessment with the use of SCORE2-Diabetes, an algorithm specifically dedicated to this group [12].

Quality of life was assessed using the standardized HeartQoL questionnaire, a tool recommended by the European Association of Preventive Cardiology (EAPC) [4,13,14,15]. The HeartQoL questionnaire consists of 14 questions that assess the quality of life in the emotional (HeartQoL emotional) and physical (HeartQoL physical) dimensions, as well as globally (HeartQoL global). In each question, you can score from 0 to 3 points. A higher score indicates a better quality of life. Quality of life in individual subscales and as a global score was calculated based on the mean scores from individual questions.

The statistical analysis was carried out using the Statistica 13.0 package (TIBCO Software Inc., Palo Alto, CA, USA). Categorical variables were expressed as the number and the percentage. Continuous variables were presented as means with standard deviations or medians with interquartile range. The Shapiro–Wilk test demonstrated a non-normal distribution of the investigated continuous variables. Therefore, non-parametric tests were used for statistical analysis. Comparisons between two groups were performed with the Mann–Whitney unpaired rank sum test. For comparisons between more groups, the Kruskal–Wallis one-way analysis of variance was used. To assess the relationship between two quantitative variables, Spearman’s rank correlation was used. To identify independent predictors of HeartQoL questionnaire results, multiple linear regression analysis was performed. Results were considered significant at *p* < 0.05.

## 3. Results

### 3.1. Cardiovascular Risk

**Figure 1:**
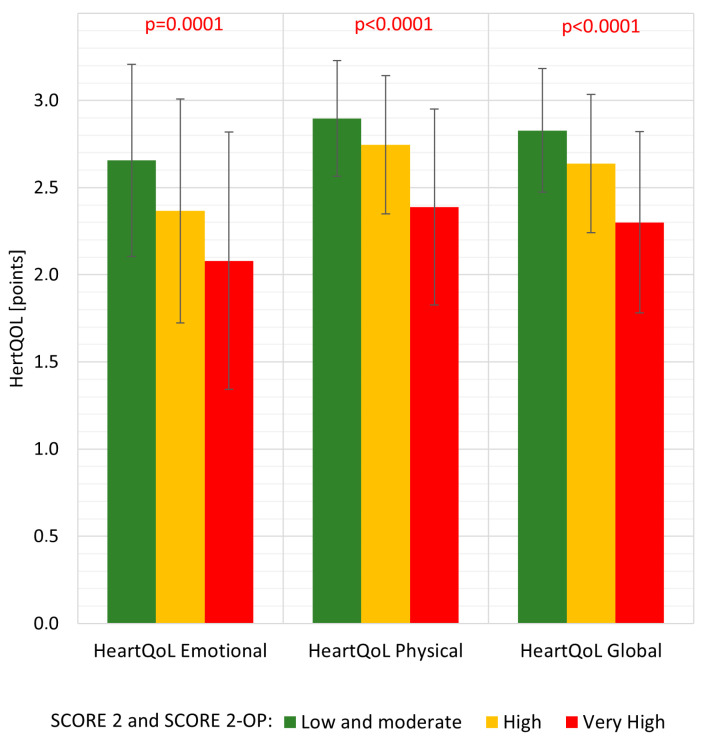
Mean quality of life assessed by the HeartQoL questionnaire (emotional and physical subscales and overall global score) in subgroups of patients with increasing total cardiovascular risk assessed with SCORE2 and SCORE2-OP algorithms.

The median 10-year CV risk assessed by the SCORE2 and SCORE2-OP algorithms was 3.0 (IQR 1.5–7.0). An increase in mean cardiovascular risk in the range from low and moderate to very high was associated with a decrease in quality of life both in individual subscales and the overall score (Figure 1). This relationship was also confirmed by Spearman’s negative correlation (HeartQoL emotional R = −0.3989, *p* < 0.0001; HeartQoL physical: R = −0.4913, *p* < 0.0001; HeartQoL global R = −0.4907, *p* < 0.0001).

### 3.2. Controlled Risk Factors

**Figure 2:**
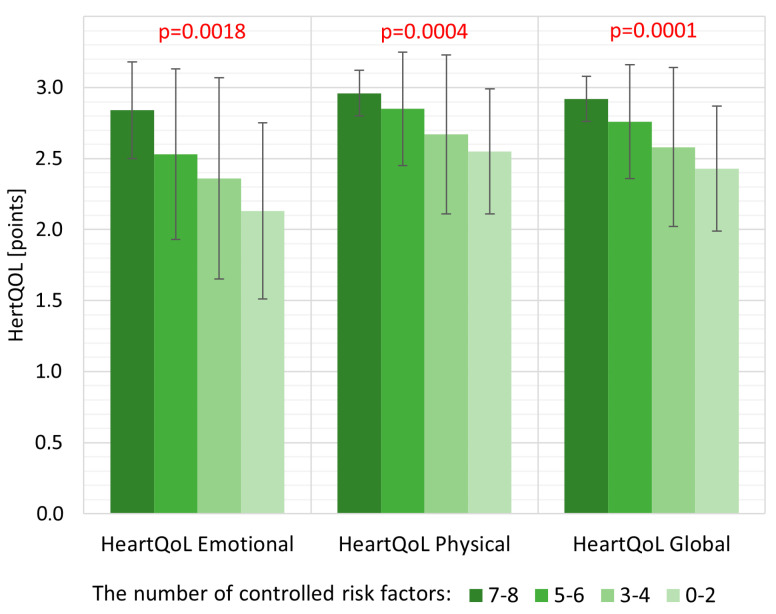
Mean quality of life assessed by the HeartQoL questionnaire (emotional and physical subscales and overall global score) in subgroups of patients with a decreasing number of controlled cardiovascular risk factors.

The median number of controlled risk factors in the study population was 4.0 (IQR 3.0–5.0). It was found that as the mean number of controlled risk factors increased, the quality of life improved in each of the studied dimensions (Figure 2).

Individuals who reported regular physical activity and who were characterized by blood glucose, arterial blood pressure, and waist circumference within the normal range had higher scores on both subscales and the overall HeartQoL score in the univariate analysis (Table 3). The other risk factors assessed separately did not differentiate the study population in terms of quality of life.

### 3.3. Reported Undertaken Health-Promoting Behaviors

**Figure 3:**
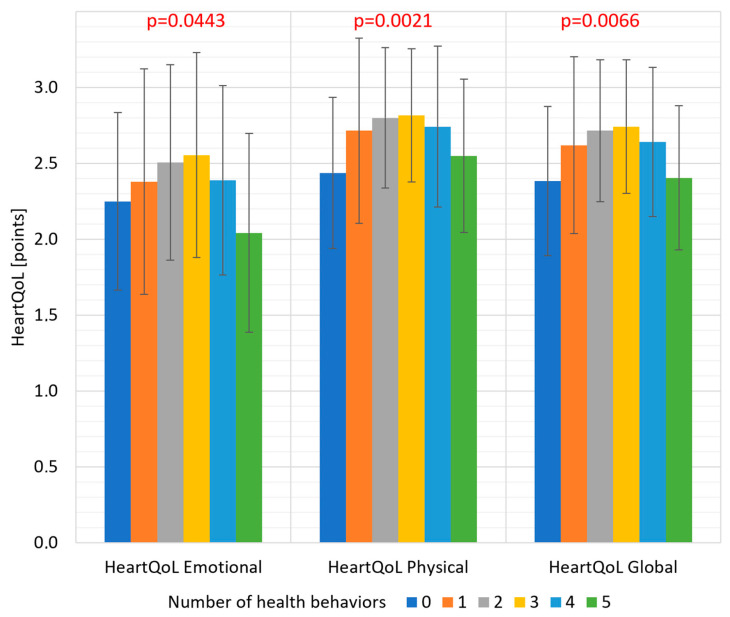
Mean quality of life assessed by HeartQoL questionnaire (emotional and physical subscales and overall global score) in subgroups of patients with an increasing number of reported health-promoting behaviors undertaken.

The median number of reported health-promoting behaviors undertaken within 3 years before study enrolment was 3.0 (IQR 2.0–4.0). The percentage of patients undertaking specific health-promoting behaviors is shown in Table 1. The highest quality of life in each of the studied dimensions was found in people who reported undertaking three health-promoting behaviors (Figure 3).

Higher quality of life was seen in those who reported dietary changes (HeartQoL physical and the overall score) and in those who reported increased physical activity (both subscales and the HeartQoL global score). On the other hand, those who reported reduced alcohol consumption had a lower quality of life based on the HeartQoL emotional subscale (Table 4).

## 4. Discussion

According to World Health Organization (WHO) reports, non-communicable diseases, including cardiovascular diseases, constitute a serious health, social, and economic problem. Taking action to eliminate modifiable risk factors for these diseases is of interest to many institutions. As one of the leading causes of disease and premature death worldwide, cardiovascular diseases require prevention as well as early diagnosis and treatment. Reducing the prevalence of non-communicable diseases and the high mortality associated with them is one of the main objectives of improving the health situation planned by the WHO for the coming years [16].

Adopting health-promoting behaviors, such as regular physical activity, changing dietary habits, or controlling blood pressure values, as well as eliminating risky behaviors such as smoking, may not only affect the health and quality of life of individual patients but also lead to beneficial social and economic consequences [17].

Some authors suggest that a higher quality of life may be associated with more effective control of CV risk factors, better functioning, and improved prognosis in chronic diseases [8,18,19,20,21,22]. There are also reports on the impact of specific risk factors on the prognosis and treatment process of ischemic heart disease [4,5,6,7,8,23,24,25,26]. The impact of chronic diseases on the patient’s well-being and quality of life depends, among other things, on the degree of disease progression and the severity of symptoms [8,27,28]. Nevertheless, to the best of our knowledge, our study is the first to answer the question of whether and to what extent achieving treatment goals for controlling CVD risk factors and undertaking health-promoting behaviors affects the quality of life of people without clinically evident atherosclerotic CVD.

In subjects without clinically evident CVD, we found that increased CV risk in the range from low and moderate to very high assessed using the SCORE2 and SCORE2-OP algorithms was associated with decreased quality of life on both the individual subscales and the overall HeartQoL score. We established that as the number of controlled risk factors increased, quality of life improved in each of the studied dimensions. The analysis demonstrated that regular physical activity, normal fasting glucose, correct waist circumference, and correct blood pressure were all independent single risk factors affecting the quality of life assessed with the HeartQoL global scale, as well as both emotional and physical subscales. This appears to be consistent with the results confirming that the control of risk factors has a positive impact on many aspects of the patient’s functioning, including quality of life [8,28,29,30]. Positive effects of physical activity on lowering systolic and diastolic blood pressure and improving quality of life have also been described [31,32]. These correlations highlight the importance and usefulness of quality of life assessment in patients with high CV risk or with clinically evident CVD. The adverse effect of increasing BMI on overall functioning and physical activity levels, especially in patients with ischemic heart disease and morbid obesity, has been previously described by A. Oreopoulos et al. [7]; however, we were unable to show an association between quality of life and BMI, while we did show such an association with waist circumference.

We found an interesting correlation between beneficial changes in health-promoting behaviors reported by patients and the HeartQoL scores. The lowest quality of life was found in patients who made no lifestyle changes at all and those who made the most lifestyle changes. The highest quality of life in each studied dimension was found in people who reported undertaking a moderate number of health-promoting behaviors. It can be assumed that taking on too many health-promoting behaviors is associated with increased pressure and stress imposed by the patient on themself. A similar relationship is observed in the group of patients suffering from orthorexia, where it is noted that increased attention to diet does not translate into better well-being and may be a source of anxiety [33]. The best quality of life was recorded in the group with an average number of health-promoting behaviors. The relationship between quality of life and beneficial health-promoting behaviors is probably two-way. On the one hand, controlling risk factors and undertaking health-promoting behaviors can improve quality of life, and on the other hand, good quality of life facilitates the implementation of expected health-promoting behaviors [34].

As expected, individual health-promoting behaviors associated with higher quality of life turned out to be increased physical activity and dietary changes. Physical activity is a factor that significantly influences many aspects of a CVD patient’s life and improves their prognosis [24,26]. Physical activity is strongly associated with the functioning of patients with ischemic heart disease [5] and aerobic exercise helps to reduce the impact of adverse risk factors and improves the declared quality of life [6], which is consistent with our results. A diet aimed at reducing excess body weight can be found in all guidelines for the prevention and treatment of cardiovascular diseases [1,2,34]. It has been proven that, as in our study group, a calorie-restricted diet [35] and the introduction of dietary rules based on the elimination of highly processed fats, the consumption of fish and seafood, and adequate amounts of fruit, vegetables, and fiber [23,25] has a beneficial effect on the reduction of risk factors and improves the quality of life of patients. Unexpectedly, reducing alcohol consumption negatively affected the emotional component of the QoL assessment in the population we studied. It is worth noting that similar results were obtained by T. Thara Govindaraju et al. [29].

Limitations of this study are the limited number of patients included and the absence of long-term follow-up, along with the imprecise definitions we used of physical activity and declared health-promoting behaviors. Furthermore, this study was also limited by a lack of complete data to assess the diabetic population using the SCORE2-Diabetes algorithm.

## 5. Conclusions

The quality of life is affected by the health-promoting behaviors undertaken, depending on their number. The highest quality of life in each of the studied dimensions was found in people who reported undertaking a moderate number of health-promoting behaviors. On the other hand, extreme behaviors, i.e., not undertaking any health-promoting behaviors or undertaking a very large number of them, were associated with a poorer quality of life.

## Figures and Tables

**Table 1:** Socio-demographic and clinical characteristics of the study group—qualitative variables.

Parameter		Entire Population		Non-Diabetic Population	
		N	%	N	%
Gender	Male	67	33.5	54	33.3
	Female	133	66.5	108	66.7
Diseases for inclusion in the study:					
Arterial hypertension	Diagnosed	127	63.5	111	68.5
Diabetes mellitus	Diagnosed	38	19.0	0	0
Hypercholesterolemia	Diagnosed	90	45.0	76	46.9
Controlled risk factors:					
Non-smoker	Declared	170	85.0	138	85.2
Systolic blood pressure	<140 mmHg	161	80.5	133	82.1
Diastolic blood pressure	<90 mmHg	179	89.5	146	90.1
Systolic/diastolic blood pressure	<140 mmHg and <90 mmHg	155	77.5	129	79.6
BMI	Underweight (<20.0 kg/m^2^)	11	5.5	11	6.8
	Correct weight (20.0–24.9 kg/m^2^)	72	36.0	62	38.3
	Overweight (25.0–29.9 kg/m^2^)	84	42.0	68	42.2
	Obesity (≥30.0 kg/m^2^)	33	16.5	21	13.0
Waist circumference	Normal fat distribution (W < 80 cm, M < 94 cm)	74	37.0	61	37.7
	Moderate central fat accumulation (W ≥ 80 cm, M ≥ 94 cm)	57	28.5	48	29.6
	High central fat accumulation (W ≥ 88 cm, M ≥ 102 cm)	69	34.5	53	32.7
Physical activity	No activity	30	15.0	23	14.2
	Low activity	110	55.0	88	54.3
	Regular activity	60	30.0	51	31.5
Serum LDL-C concentration	<2.6 mmol/L	46	23.0	36	22.2
Serum TG concentration	<1.7 mmol/L	163	81.5	137	84.5
Fasting plasma glucose	<5.56 mmol/L	117	58.5	104	64.2
Number of controlled measures of CV risk factors	0	2	1.0	1	0.6
	1	5	2.5	3	1.9
	2	15	7.5	11	6.8
	3	43	21.5	33	20.4
	4	52	26.0	40	24.7
	5	35	17.5	32	19.8
	6	32	16.0	27	16.7
	7	10	5.0	9	5.6
	8	6	3.0	6	3.7
Number of reported health-promoting behaviors	0	16	8.0	14	8.6
	1	25	12.5	23	14.2
	2	43	21.5	39	24.1
	3	59	29.5	49	30.2
	4	45	22.5	32	19.8
	5	12	6.0	5	3.1
Estimated cardiovascular risk (SCORE2 and SCORE2-OP)	Low or moderate	NA	NA	96	59.3
	High	NA	NA	41	25.3
	Very high	NA	NA	25	15.4

Note: NA—not applicable.

**Table 2:** Socio-demographic and clinical characteristics of the study group—quantitative variables.

Parameter	Entire Population (N = 200)		Non-diabetic Population (N = 162)	
	Mean (±SD)	Median (IQR)	Mean (±SD)	Median (IQR)
Age	51.6 (±13.6)	52.0 (43.0–60.5)	50.4 (±13.6)	51.0 (41–59)
Systolic blood pressure (mmHg)	127.2 (±14.5)	125.0 (118.0–135.0)	126.6 (±14.0)	125.0 (118.0–134.0)
Diastolic blood pressure (mmHg)	76.5 (±9.3)	77.5 (70.0–82.0)	76.4 (±9.1)	76.0 (70.0–82.0)
Waist circumference (cm)	88.0 (±12.2)	87.0 (80.0–95.5)	87.2 (±11.9)	86.0 (79.0–94.0)
BMI (kg/m^2^)	26.4 (±4.1)	26.0 (23.9–28.7)	26.0 (±3.7)	25.9 (23.9–28.1)
Serum total cholesterol concentration (mmol/L)	5.6 (±1.1)	5.6 (4.9–6.3)	5.6 (±1.0)	5.6 (5.0–6.2)
Serum HDL-C concentration (mmol/L)	1.6 (±0.4)	1.5 (1.3–1.8)	1.6 (±0.4)	1.5 (1.3–1.8)
Serum non-HDL-C concentration (mmol/L)	4.1 (±1.0)	4.0 (3.4–4.7)	4.0 (±1)	4.0 (3.3–4.6)
Serum LDL-C concentration (mmol/L)	3.3 (±1.0)	3.3 (2.7–4)	3.3 (±0.9)	3.3 (2.7–4)
Serum TG concentration (mmol/L)	1.4 (±0.8)	1.2 (0.9–1.6)	1.3 (±0.8)	1.2 (0.9–1.5)
Fasting plasma glucose (mmol/L)	6.0 (±1.2)	5.9 (5.4–6.4)	5.8 (±0.8)	5.8 (5.4–6.2)

**Table 3:** Mean quality of life in relation to the control of individual risk factors assessed by the HeartQoL questionnaire (emotional and physical subscales and overall global score).

Controlled Risk Factors	Achieving Control	N	HeartQoL Emotional		HeartQoL Physical		HeartQoL Global	
			Mean (±SD)	*p*	Mean (±SD)	*p*	Mean (±SD)	*p*
Ideal blood pressure	No	45	2.24 (±0.66)	0.0117	2.54 (±0.58)	0.0002	2.46 (±0.53)	0.0002
	Yes	155	2.48 (±0.66)		2.79 (±0.47)		2.71 (±0.47)	
Ideal waist circumference	No	126	2.33 (±0.71)	0.0123	2.67 (±0.56)	0.0083	2.57 (±0.53)	0.0026
	Yes	74	2.6 (±0.54)		2.85 (±0.36)		2.78 (±0.37)	
Ideal BMI	No	118	2.42 (±0.65)	0.6129	2.71 (±0.54)	0.8032	2.63 (±0.52)	0.5849
	Yes	72	2.44 (±0.69)		2.76 (±0.46)		2.67 (±0.46)	
Normal LDL cholesterol level	No	154	2.42 (±0.67)	0.7404	2.73 (±0.52)	0.6533	2.64 (±0.5)	0.7718
	Yes	46	2.46 (±0.65)		2.76 (±0.45)		2.68 (±0.47)	
Normal triglyceride levels	No	37	2.36 (±0.61)	0.2912	2.80 (±0.33)	0.7655	2.68 (±0.34)	0.3786
	Yes	163	2.44 (±0.68)		2.72 (±0.53)		2.64 (±0.52)	
Normal fasting glucose	No	83	2.3 (±0.67)	0.0056	2.68 (±0.50)	0.0066	2.57 (±0.48)	0.0013
	Yes	117	2.53 (±0.65)		2.78 (±0.51)		2.71 (±0.49)	
Non-smoker	No	30	2.36 (±0.82)	0.9151	2.62 (±0.55)	0.2451	2.55 (±0.59)	0.6226
	Yes	170	2.44 (±0.63)		2.76 (±0.49)		2.67 (±0.47)	
Regular physical activity	No	140	2.34 (±0.69)	0.0029	2.66 (±0.55)	<0.0001	2.57 (±0.53)	0.0001
	Yes	60	2.64 (±0.54)		2.92 (±0.30)		2.84 (±0.30)	

**Table 4:** Mean quality of life in relation to the individual health-promoting behaviors undertaken, as assessed by the HeartQoL questionnaire (emotional and physical subscales and overall global score).

Health-Promoting Behavior	Declared	N	HeartQoL Emotional		HeartQoL Physical		HeartQoL Global	
			Mean (±SD)	*p*	Mean (±SD)	*p*	Mean (±SD)	*p*
Dietary changes	No	39	2.30 (±0.69)	0.1156	2.66 (±0.45)	0.0269	2.56 (±0.47)	0.0399
	Yes	161	2.46 (±0.66)		2.76 (±0.51)		2.68 (±0.43)	
Weight reduction	No	98	2.40 (±0.71)	0.7439	2.71 (±0.55)	0.9989	2.62 (±0.54)	0.9989
	Yes	102	2.46 (±0.62)		2.77 (±0.46)		2.68 (±0.43)	
Increased physical activity	No	56	2.25 (±0.69)	0.0085	2.58 (±0.58)	0.0011	2.48 (±0.55)	0.0014
	Yes	144	2.50 (±0.64)		2.88 (±0.46)		2.71 (±0.45)	
Smoking cessation	No	10	2.28 (±0.97)	0.7601	2.29 (±0.89)	0.2020	2.29 (±0.85)	0.6115
	Yes	63	2.26 (±0.73)		2.67 (±0.51)		2.55 (±0.51)	
Decreased alcohol consumption	No	142	2.51 (±0.64)	0.0096	2.75 (±0.49)	0.1392	2.68 (±0.47)	0.0525
	Yes	58	2.25 (±0.70)		2.70 (±0.55)		2.57 (±0.53)	

## Data Availability

The data presented in this study are available from all authors.
